# On-Arrival Lumbar Epidural Analgesia for Lower Limb Trauma Pain in Emergency Care: A Superiority Randomized Controlled Trial

**DOI:** 10.7759/cureus.54622

**Published:** 2024-02-21

**Authors:** Anshul Jain, Shivali Pandey, Shivanand Sonakar, Paras Gupta, Rachna Chaurasia, Neeraj Banoria

**Affiliations:** 1 Department of Anesthesiology, Maharani Laxmi Bai Medical College, Jhansi, IND; 2 Department of Orthopedics, Maharani Laxmi Bai Medical College, Jhansi, IND; 3 Department of Radiodiagnosis, Maharani Laxmi Bai Medical College, Jhansi, IND; 4 Department of Surgery, Maharani Laxmi Bai Medical College, Jhansi, IND

**Keywords:** visual analog scale, lower limb fracture, lumbar epidural, stress response, limb fracture pain solutions, emergency pain relief, trauma epidural, trauma pain

## Abstract

Background: Lower limb trauma in emergency settings often leads to pain management challenges. Traditional methods, primarily non-steroidal anti-inflammatory drugs (NSAIDs) and opioids, have limitations. This study explores the efficacy of epidural analgesia in emergency for lower limb trauma patients (ELETRA) as an alternative for managing pain in lower limb trauma patients upon arrival in emergency care.

Objectives: This study primarily focuses on determining ELETRA's effectiveness in reducing pain for patients with lower limb trauma upon arrival. It also aims to evaluate ELETRA's influence on decreasing stress and shortening hospital stays compared to traditional pain management approaches.

Methods: This study was executed as a prospective, parallel-design, randomized controlled trial in the emergency department of a tertiary care teaching hospital. The interventions were performed in a dedicated induction room adjacent to the emergency operating theater. The trial was registered in the Clinical Trial Registry of India with registration number CTRI/2022/08/044699 dated 16/08/2022. Participants were 18- to 50-year-old patients with lower limb injuries, classified under American Society of Anesthesiologists (ASA) class 1 or 2. Participants were randomized into two groups: one receiving ELETRA and the other standard pain control treatment. The effectiveness of pain relief was measured through a visual analog scale (VAS), and hemodynamic parameters, adverse effects, levels of acute phase reactants, and stress hormones were also measured along with patient satisfaction.

Results: The study enrolled 356 participants with lower limb injuries. After excluding participants lost following the intervention, data from 157 individuals in Group A and 160 in Group B were analyzed. Group A's success rate for analgesia (VAS < 2) was 92.35% (n = 145), significantly higher than Group B's 75.62% (n = 121) (p < 0.001). The commonest side effect was hypotension (5.73%) in Group A and nausea in Group B (7.5%). C-reactive protein (CRP) levels rose to 104.71 ± 8.99 mg/dL in Group A and 192.58 ± 9.23 mg/dL in Group B; the difference was statistically significant. Serum cortisol levels were also higher in Group B (67.18 ± 9.21) compared to Group A (44.72 ± 6.14) at one week. Group B had a longer hospital stay, averaging 12.24 ± 4.81 days, against Group A's 10.19 ± 4.91 days.

Conclusion: ELETRA is a safe and effective alternative for pain management in lower limb trauma patients in emergency settings. It reduces pain, improves patient satisfaction, and has a favorable impact on stress responses.

## Introduction

The global public health landscape is increasingly burdened by trauma, standing alongside infectious and chronic diseases as a significant concern [1]. With road traffic incidents (RTIs) forecasted to ascend into the top five causes of mortality worldwide by 2030, their impact is undeniable [2,3]. The World Health Organization estimates an alarming 1.35 million fatalities annually due to RTIs, with an additional 20-50 million individuals sustaining non-lethal injuries [4].

Among non-fatal injuries, those affecting the lower limbs are predominant, leading to the majority of lower limb bone fractures [5,6]. Pain, as a prevalent presenting complaint among these patients, has profound implications, contributing to hemodynamic instability and shock and often resulting in patient dissatisfaction due to suboptimal management in emergency settings [7,8]. Traditional management in emergency care has largely been confined to non-steroidal anti-inflammatory drugs (NSAIDs) and opioids of moderate potency [9].

Regional anesthesia techniques stand out as a promising alternative, particularly for severe pain in cases of long bone fractures. They not only provide acute pain relief but also mitigate the risks associated with the use of NSAIDs and opioids, and may have a role in reducing the transition to chronic pain [9].

The success of neuraxial blocks in managing the intense pain of labor, comparable to or exceeding that of fracture pain, provides a strong argument for their potential in trauma care [10]. Epidural analgesia, especially when using diluted local anesthetics, effectively manages labor pain while maintaining muscular function, suggesting benefits for lower limb trauma patients. This approach could offer dual advantages: maintaining motor function to support venous return via the calf muscles, thereby potentially diminishing the risk of thrombotic events and shortening hospital admissions.

This investigation introduces epidural analgesia in emergency for lower limb trauma patients (ELETRA) as a novel application of epidural analgesia in the context of lower limb trauma. The primary aim of this study is to evaluate the efficacy of ELETRA in alleviating pain upon arrival for patients with lower limb trauma. Secondary aims include assessing ELETRA's impact on reducing stress and the potential to curtail the length of hospitalization compared to conventional pain management methods.

## Materials and methods

The study was executed as a prospective, parallel-group, randomized controlled trial with a 1:1 allocation ratio at a tertiary care teaching hospital. All experimental protocols and procedures received approval from the institutional ethics committee of Maharani Laxmi Bai Medical College (approval number: 1153/IEC/2021, dated 02/06/2022) and adhere to the 2018 Declaration of Helsinki guidelines. Informed and written consent was obtained from all participants concerning their participation and the use of their data for this research. The trial was prospectively registered in the Clinical Trial Registry of India (registration number: CTRI/2022/08/044699, dated 16/08/2022). The methods of this study were structured in accordance with the CONSORT reporting guidelines [11].

Participants were individuals between 18 and 50 years of age with lower limb injuries, assessed under the American Society of Anesthesiologists (ASA) class 1. Upon arrival, trauma victims were received in the triage area of the emergency department. Following a primary survey and a negative focused assessment with sonography for trauma (FAST) scan, patients were given injection diclofenac 75 mg as a rescue analgesic in accordance with institutional protocol. Point-of-care testing for coagulopathy prothrombin time (PT) along with international normalized ratio (INR) and activated partial thromboplastin time (aPTT) was performed to rule out bleeding disorders.

The study excluded patients who refused to participate, were unable to consent, had associated upper limb trauma or polytrauma, or met any of the following criteria: a history of allergy to any of the study medications, coagulation abnormality (INR > 1.5) or on anticoagulant therapy, hemoglobin level less than 10 g/dL, hemodynamic instability (heart rate > 120 beats per minute or blood pressure < 100/60 mmHg), and pregnancy.

Site of the study

The study was executed in the emergency department; interventions were performed in a dedicated induction room adjacent to the emergency operating theater, which was fully equipped with all necessary resuscitation and monitoring facilities. After an initial 30-minute period post-intervention, participants were transferred to their respective beds where the outcomes were measured using designated scales and blood samples collected bedside.

Randomization and intervention

In the current study, cases were allocated to either Group A or Group B by an independent researcher using random allocation cards, which were created based on computer-generated random numbers, to ensure the elimination of selection bias.

Patients in Group A received epidural analgesia, and patients in Group B received standard-of-care treatment for pain control.

For Group A, epidural analgesia was initiated, utilizing 0.2% ropivacaine at a dosage of 2 mg/mL. The technique involved the insertion of a 20G epidural catheter through an 18G Tuohy needle, applied with strict aseptic precautions at the L4-L5 or L3-L4 interspace. The catheter was threaded 3-5 cm into the epidural space. An initial bolus of 10 mL of the ropivacaine solution was administered, following verification with a test dose. Maintenance of analgesia was achieved via a patient-controlled epidural analgesia pump, which delivered 2 mL boluses with a 30-minute lockout period and a steady background infusion set at 6 mL/hour. Hemodynamic parameters were continuously monitored.

Conversely, Group B received the standard treatment for managing pain. This regimen was in accordance with the established institutional protocol, which included intramuscular diclofenac 75 mg twice daily and injection tramadol 50 mg bolus intramuscularly. This was followed by intravenous infusion of tramadol through a patient-controlled analgesia (PCA) mechanism providing a 5 mg/hour (1 mg/mL) basal infusion, a 2 mg bolus option with a 30-minute lock-in period, and a maximum limit of 50 mg over a six-hour interval.

In situations where primary surgical intervention was planned, a combined spinal-epidural modality was introduced. For individuals in Group A, this meant an additional intrathecal block at the L2-L3 or L3-L4 interspace using a mixture of 0.5% levobupivacaine (2 mL) and fentanyl (50 mg), for a total volume of 3 mL. Group B's approach of combined spinal epidural involved a two-prick technique, employing an 18G epidural needle alongside a 23G spinal needle. The intrathecal dose was the same as that of Group A; block was extended using epidural ropivacaine 0.2% in the dosage of 6-8 mL per hour. Postoperatively, analgesia was managed with a patient-controlled epidural analgesia system, dispensing a solution of 0.625% levobupivacaine with fentanyl (5 mg/mL) in both groups.

Outcomes

The effectiveness of analgesia was appraised using the visual analog scale (VAS) [12]. A VAS score of 2 or less was indicative of initial success. Participants who did not attain a score of 2 or below within one hour post-intervention were classified as non-responders. Adverse hemodynamic incidents were characterized by the following parameters. Hypotension was defined as a systolic blood pressure falling below 100 mmHg or experiencing a drop of 30% or more from the baseline. Tachycardia was identified when the heart rate exceeded 100 beats per minute. Additional adverse outcomes, including nausea/vomiting and allergic reactions, were recorded and analyzed. Satisfaction levels regarding the pain management process in the emergency care setting were measured upon patient discharge, utilizing a 5-point Likert scale [13]. The comparative stress response between the two groups was assessed by measuring acute phase reactants C-reactive protein (CRP), interleukin-6 (IL-6), and D-dimer, and the stress hormone, serum cortisol. Levels of serum CRP and IL-6 were ascertained through the enzyme-linked immunosorbent assay (ELISA) method and were evaluated at baseline, 12 hours, 24 hours, on the third day, and one week following the intervention (for patients still hospitalized). D-dimer levels followed the same schedule using the Clearview Simplify D-dimer assay. Serum cortisol was similarly measured at the aforementioned intervals using ELISA. The schedule of assessments is further detailed in Table 1.

**Table 1:** Schedule of clinical study procedures and assessments a: Coagulation studies including PT, PPT, INR, and platelet count were estimated by point-of-care testing. b: APRs IL-6, CRP, and D-dimer were measured. c: Measured at 48 hours post-intervention. d: Standard of care. VAS: visual analog scale, ECG: electrocardiogram, PT: prothrombin time, PTT: partial thromboplastin time, INR: international normalized ratio, APR: acute phase reactants, IL-6: interleukin-6, CRP: C-reactive protein, SOC: standard of care

Procedure	Screening	Baseline	6 hours	12 hours	24 hours	36-48 hours	48-72 hours	1 week	Discharge
Inclusion/exclusion	X	X		X					
Informed consent	X								
Demographics	X								
Medical history	X								
Physical examination including ECG	X				X		X		
Hemoglobin and coagulation profile^a^	X				X		X		
Sampling for CRP^b^, IL-6, and D-dimer		X		X	X	X^c^	X		
Sampling for serum cortisol		X			X		X	X	
Randomization									
Intervention (epidural analgesia or SOC^d^)			X	X	X	X	X		
Adverse events				X	X		X	X	
VAS scoring				X	X	X	X	X	
Likert scoring									X

Statistical analysis

The requisite sample size for the study was calculated using a dedicated sample size calculator tailored for superiority trials [14]. With an aim to establish a statistically significant difference in treatment efficacy, an anticipated difference of 2 points in pain scores was used, with an assumed standard deviation (SD) of 3.5. Adopting a two-sided type I error rate of 0.05 and seeking a study power of 0.85, the initial sample size computation indicated the need for 256 participants, distributed evenly between the two study arms. To pre-empt and adjust for possible attrition, as well as variability and scenarios such as participants transferring out post-intervention, the sample size was judiciously augmented to 356 individuals. For the analysis, data were represented in terms of mean, frequency, and percentage. Dichotomous variables were detailed as counts/frequencies and examined using the Chi-square test. Continuous variables were displayed as mean ± SD and assessed for differences using independent "t" tests when comparing the two groups. The Statistical Package for the Social Sciences (SPSS) software for MAC OS version 22 (IBM SPSS Inc., Chicago, IL) was employed for all statistical testing. Results yielding a p-value of less than 0.05 were considered statistically significant, with a 95% confidence interval being the standard benchmark for this determination.

## Results

**Figure 1:**
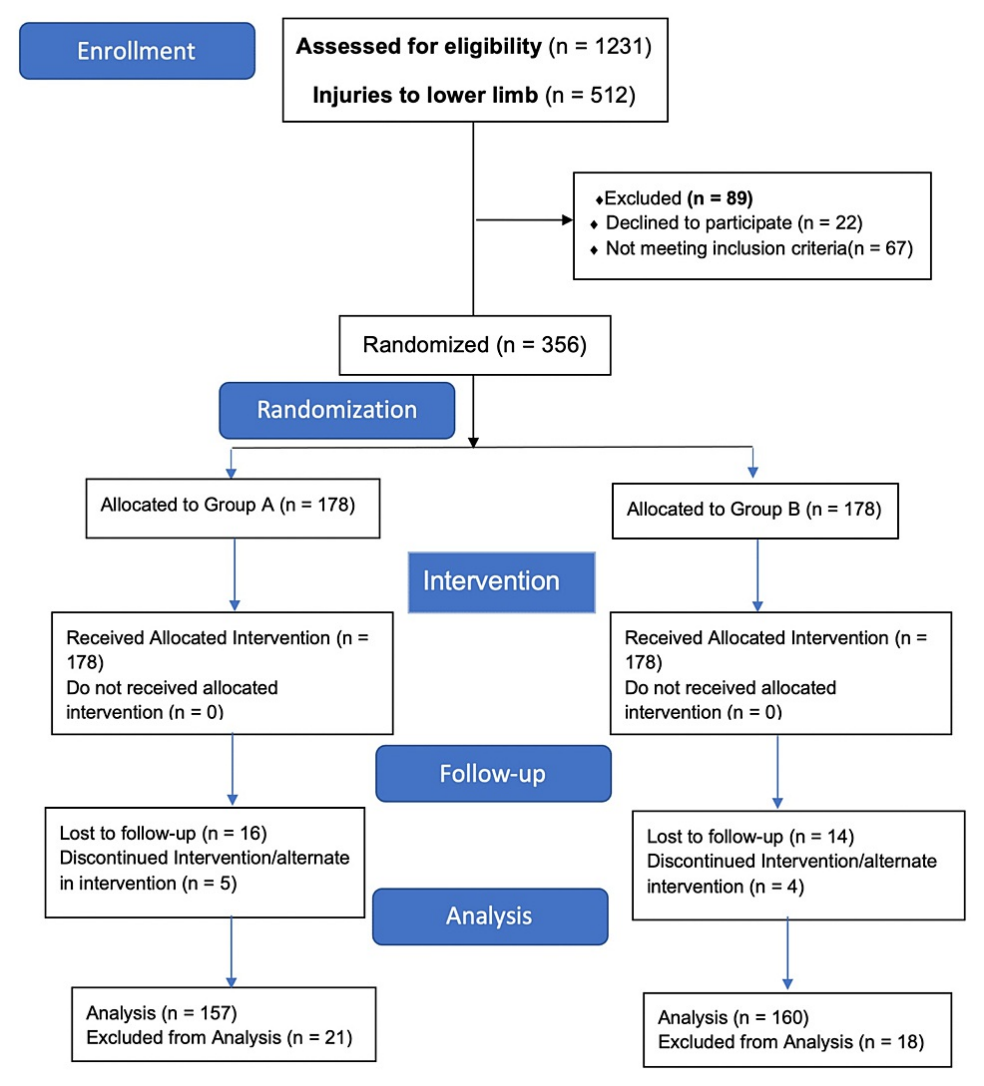
CONSORT flow diagram

The investigation spanned from October 2022 to November 2023. Within this timeframe, 1,231 patients admitted to the casualty department due to trauma were reviewed, with 512 presenting injuries confined to the lower limbs. A thorough analysis identified 423 patients who met the inclusion criteria. After applying the exclusion criteria, 67 patients were deemed ineligible, culminating in a final tally of 356 participants who underwent randomization as depicted in the CONSORT flow diagram (Figure 1).

Recruitment in the trial ceased on November 16, 2023, once the predesignated sample size was met. Post-randomization, 39 individuals were withdrawn for reasons outlined in the CONSORT flow diagram, leading to 157 participants in Group A and 160 in Group B being subject to data analysis. Demographics and injury distribution, as summarized in Table 2, were statistically analogous between the groups, with femur fractures emerging as the most common injury, followed by concurrent tibia and fibula fractures. Major surgical intervention was performed within the first 24 hours of injury in 136 participants (Group A: 71, Group B: 65) and 89 participants (Group A: 46, Group B: 43) in the next 24 hours. Seventy-two participants (Group A: 34, Group B: 38) were managed by minor surgical procedure (closed reduction) or non-surgically by slab application, and statistically, there was no significant difference.

**Table 2:** Demographic and injury profile of the participants n: number of patients, SD: standard deviation, BMI: body mass index

Variable	Group A (n = 157)	Group B (n = 160)	p-value
Age (mean ± SD)	37.29 ± 7.32	38.22 ± 8.53	0.0716
Male:female	93:64	89:71	0.591
Height (in meters) (mean ± SD)	1.52 ± 0.21	1.48 ± 0.23	0.620
Weight (in kg)(mean ± SD)	72.28 ± 9.83	73.65 ± 8.51	0.283
BMI (kg/m^2^) (mean ± SD)	29.56 ± 5.87	28.18 ± 6.94	0.27
Injury distribution			
Femur fracture (n)	43	41	0.79
Tibia fracture (n)	25	28	0.76
Tibia and fibula fracture (n)	36	39	0.792
Foot bone fracture (n)	21	18	0.61
Combined tibia and femur fracture (n)	10	8	0.63
Other (n)	22	26	0.63
Admission to intervention time (in minutes) (mean ± SD)	66.34 ± 12.21	63.94 ± 11.23	0.06
Surgical intervention			
First 24 hours	71	65	0.09
24-48 hours	46	43	0.71

A total of 266 (Group A: 145, Group B: 121) patients reported sufficient analgesia (VAS < 2). The success rate was calculated to be 92.35% in Group A and 75.62% in Group B. The difference was found to be highly significant (Table 3).

**Table 3:** Group-based trends in VAS scores and hemodynamic parameters post-intervention over time ^#^Statistically significant VAS: visual analog scale, MAP: mean arterial pressure

	Parameters								
	VAS score			Heart rate			MAP		
	Group A (n = 157)	Group B (n = 160)	p-value	Group A (n = 157)	Group B (n = 160)	p-value	Group A (n = 157)	Group B (n = 160)	p-value
Baseline (at admission)	8.23 ± 1.3	8.11 ± 1.22	0.54	102.12 ± 12.37	99.61 ± 12.41	0.071	89.23 ± 13.12	91.16 ± 12.91	0.17
Just before intervention	6.41 ± 2.01	5.99 ± 1.99	0.09	99.18 ± 7.87	97.38 ± 9.23	0.065	87.43 ± 12.25	89.17 ± 13.23	0.24
1 hour post-intervention	1.91 ± 0.89	2.96 ± 1.32	<0.001^#^	91.53 ± 7.23	94.42 ± 11.31	0.007^#^	82.36 ± 14.89	91.05 ± 12.17	<0.0001^#^
6 hours (post-intervention)	1.71 ± 1.05	3.21 ± 1.96	<0.001^#^	86.48 ± 8.91	91.61 ± 8.32	<0.001^#^	83.27 ± 14.13	92.26 ± 13.12	<0.001^#^
12 hours	1.97 ± 0.91	3.51 ± 0.96	<0.001^#^	82.67 ± 7.68	89.83 ± 7.21	<0.0001^#^	85.33 ± 12.56	93.21 ± 12.94	<0.001^#^
24 hours	1.83 ± 0.63	3.23 ± 1.39	<0.001^#^	83.12 ± 8.12	87.45 ± 8.93	<0.0001	86.84 ± 12.79	92.29 ± 13.85	<0.001^#^
36 hours (left over participants)	1.98 ± 0.87	3.12 ± 1.61	<0.001^#^	84.34 ± 7.83	86.57 ± 7.81	0.15	91.34 ± 14.36	93.37 ± 13.68	0.198
48 hours (left over participants)	1.88 ± 0.98	3.34 ± 1.12	<0.001^#^	82.25 ± 7.12	83.79 ± 9.21	0.40	92.45 ± 13.34	93.89 ± 14.51	0.358

Following the intervention in Group A, a statistically significant decrease was observed in both heart rate (99.18 ± 7.87 to 91.53 ± 7.23) and mean arterial pressure (89.23 ± 13.12 to 82.36 ± 14.89). Notably, only 11 participants in Group A met the criteria for hypotension, yielding an incidence of 7.66%. In contrast, Group B exhibited a persistently elevated heart rate after intervention, with no significant alteration in mean arterial pressure among its participants. The analysis of adverse effects indicated that hypotension was the most prevalent side effect in Group A, affecting 5.73% of participants, while nausea was the most common side effect in Group B, occurring in 7.5% of participants. Importantly, no serious adverse side effects were observed in either group (Table 4).

**Table 4:** Groupwise distribution of adverse effects ^#^Statistically significant HR: heart rate, bpm: beats per minute

Adverse effect	Group A (n = 157)	Group B (n = 160)	p-value
Tachycardia (HR > 100 bpm)	11	21	0.105
Hypotension	12	2	0.0058^#^
Bradycardia	0	1	1.0
Palpitation	7	13	0.266
Arrhythmias	0	0	Not applicable
Nausea	3	23	0.000046^#^
Vomiting	1	7	0.067
Constipation	3	8	0.219
Shivering	9	1	0.01^#^
Headache	1	2	1.0
Backache	4	0	0.059
Numbness	3	0	0.120

The mean CRP level in Group A was 11.89 ± 5.21 mg/dL at baseline, peaking to 104.71 ± 8.99 mg/dL at 24 hours. For Group B, the mean CRP level was 13.10 ± 6.87 mg/dL at baseline, increasing to 192.58 ± 9.23 mg/dL at 24 hours. Group B exhibited a higher mean CRP level post-intervention compared to Group A over the study duration. The mean IL-6 level in Group A was 25.56 ± 7.12 pg/mL at baseline, escalating to 68.51 ± 5.18 pg/mL at 24 hours. In Group B, it started at 26.89 ± 5.13 pg/mL and reached 92.39 ± 7.12 pg/mL at 24 hours, indicating a more significant increase in Group B. At one week, the levels of IL-6 were comparable between both groups. Serum cortisol levels showed a gradual rise in both groups, with a notably higher elevation in Group B at the one-week mark, reaching 31.89 ± 4.22 μg/dL. The mean D-dimer levels in both groups exhibited a gradual and comparable increase (Table 5).

**Table 5:** Analysis of stress hormone responses between Group A and Group B over time following intervention ^#^Statistically significant IL-6: interleukin-6, CRP: C-reactive protein

Time point	Parameter	Group A (n = 157)	Group B (n = 160)	p-value
Just before intervention	IL-6 (pg/mL)	25.56 ± 7.12	26.89 ± 5.13	0.056932
	CRP (mg/dL)	11.89 ± 5.21	13.10 ± 6.87	0.078625
	Serum cortisol (μg/dL)	17.23 ± 3.23	16.65 ± 2.21	0.062564
12 hours	IL-6	34.51 ± 6.17	39.72 ± 8.24	<0.001 ^#^
	CRP	76.69 ± 4.36	126.92 ± 9.56	<0.001^#^
	Serum cortisol	22.29 ± 4.51	23.31 ± 6.24	0.087^#^
24 hours	IL-6	68.51 ± 5.18	92.39 ± 7.12	<0.001
	CRP	104.71 ± 8.99	192.58 ± 9.23	<0.001
	Serum cortisol	26.46 ± 5.25	28.27 ± 7.28	0.053
3rd day (72-78 hours)	IL-6	49.41 ± 6.12	71.49 ± 7.13	0.01
	CRP	56.29 ± 7.65	89.56 ± 8.51	<0.001
	Serum cortisol	29.72 ± 3.12	37.34 ± 5.29	<0.001
1 week (n = 101)	IL-6	29.41 ± 6.12	31.49 ± 7.13	0.122
	CRP	30.21 ± 7.65	34.56 ± 9.51	0.008
	Serum cortisol	44.72 ± 6.14	67.18 ± 9.21	<0.001

**Figure 2:**
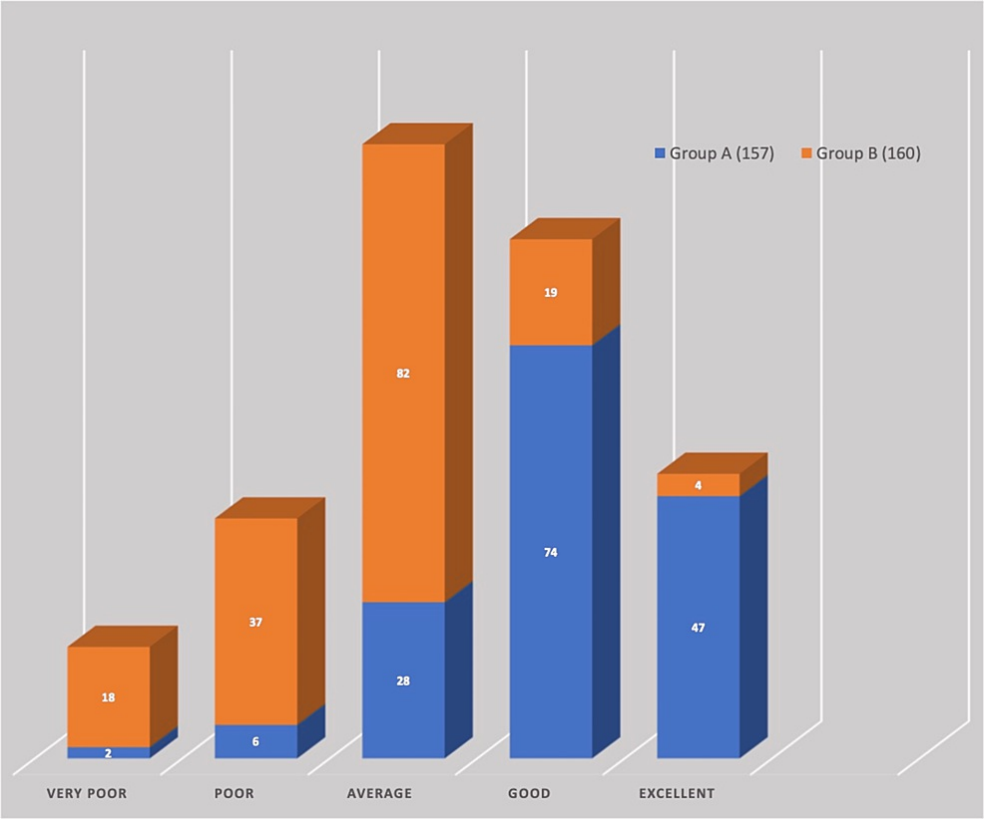
Comparative analysis of patient satisfaction with pain control techniques in the emergency area using Likert scale ratings at discharge

The duration of stay in the hospital was higher in Group B (mean: 12.24 ± 4.81, range: 3-39 days) compared to Group A (mean: 10.19 ± 4.91, range: 4-31 days). At the time of discharge, the participants were also evaluated for the satisfaction of the pain control technique in the emergency area with the Likert scale; there was a significant difference in the patients who reported poor (Group A: 2, Group B: 18) and excellent ratings (Group A: 47, Group B: 4) (Figure 2).

## Discussion

Effective analgesia constitutes a critical objective in emergency medical care from a patient's perspective. Nevertheless, data reveals that merely half of trauma patients are administered analgesics, with a majority experiencing suboptimal pain relief [15]. This trend is observed regardless of the healthcare professional's role in administering the analgesic. Predominantly, apprehensions regarding adverse effects and ambiguities in determining appropriate dosages contribute to this inadequacy in analgesia provision. In the current study, the authors evaluated ELETRA for the pain control of traumatic lower limb injury in the emergency area. According to the findings, epidural analgesia provides better pain control in comparison to systemic medications. As per our knowledge, the current study is the first global study to evaluate epidural analgesia for pain control in lower limb trauma patients in the emergency department.

The researchers favored ropivacaine as local anesthetic in the current study, owing to its distinctive differential block property that preferentially preserves motor function while minimizing hypotension. This characteristic, along with its lower potential for central nervous system and cardiotoxicity, and its effectiveness in producing a prolonged sensory blockade, positions ropivacaine as a preferable choice for the current study [16].

In the current study, the authors used a 0.2% concentration of ropivacaine on the basis of previous studies, which concluded 0.2% as a safe dose for epidural analgesia [17]. By using a low dose, firstly, the authors alleviate local anesthetic toxicity. Secondly, in low doses, motor fibers remain spared, which reduces the intensity of hypotension by preserving the pumping action of the calf muscles. Thirdly, preserving motor activity also reduces the chances of blood stasis and therefore the risk of venous thrombosis. The chosen infusion dose of 6 mL/hour for the study was based on previous research, which suggested that this rate is potentially the most effective minimal dosage, offering an optimal balance between pain alleviation and motor block [18].

In the current research, hypotension was the commonest side effect in the patients subjected to epidural analgesia, whereas nausea was the commonest side effect in patients subjected to standard of care. However, this reduction was not precipitous, and significant hypotension requiring vasopressor was seen in only a minority of cases. Hypotension is a prevalent physiological response to epidural and spinal anesthesia, primarily induced by sympathetic nervous system blockade, which causes arterial and venous vasodilation, leading to "functional" hypovolemia [19]. The attenuated blood pressure drop in the current study may be ascribed to the administration of a diluted anesthetic agent, which sufficiently preserves motor functions, as reported in multiple studies where epidural analgesia was used for labor pain [17,20].

In the current study, the authors adopted the VAS score for the evaluation of pain. The visual analog scale (VAS) is a pain rating scale first used by Hayes and Patterson in 1921 [12]. Since then, the VAS score has been used successfully for the assessment of acute pain. The authors utilized the Likert scale for the evaluation of the pain control method in the emergency area; however, the authors performed this evaluation at the time of discharge. Evaluating pain control satisfaction in the emergency area at the time of discharge provides critical insights into the effectiveness and patient-centeredness of the analgesic techniques used. This timing allows for a comprehensive assessment of the entire pain management process, reflecting both the immediate and sustained efficacy of the intervention. This approach aligns with the goal of continuous quality improvement in emergency care, ensuring that pain management strategies are both effective and aligned with patient expectations and comfort. The finding of the current study aligns with previous studies, which also reported that epidural analgesia was linked with better patient comfort [21,22].

In the current study, the authors evaluated stress response by assessing CRP, IL-6, D-dimer, and serum cortisol levels. IL-6 is a multifunctional cytokine involved in regulating immune and inflammatory responses. Recent clinical studies have explored IL-6's response to trauma, burns, and elective surgery, highlighting its significance in medical and healing processes [23]. IL-6 is under negative control by glucocorticoids [24]. The finding of the current study reveals a rise in IL-6 in both groups; the peak value was however more in Group B. Higher peak values in Group B can be attributed to poor pain control in that group. Indra et al. in a prospective study concluded a positive correlation between IL-6 level and postoperative pain, signifying higher IL-6 level in the presence of pain [24].

CRP is a pentameric protein synthesized by the liver, whose level rises in response to inflammation. CRP is an acute phase reactant protein that is primarily induced by IL-6. C-reactive protein (CRP) levels can also be related to acute pain. Acute pain, especially when caused by an inflammatory process, trauma, or infection, can lead to elevated CRP levels in the blood [25]. The results of the present study indicate a significant elevation in CRP levels in Group B, which exhibited suboptimal pain management. Previously, many studies reported higher CRP in the context of severe pain [26,27].

In the current study, the authors also evaluated the level of serum cortisol. Serum cortisol, a gold standard stress hormone, is released by the adrenal glands in response to stress [28]. Monitoring cortisol levels provides insights into an individual's stress response and overall well-being. The current study reveals increased serum cortisol at one week in the participants with better pain control. This finding aligns with a study conducted by Özmen et al., who reported lower free serum cortisol levels in patients who achieved better pain control after cardiac surgery [29]. In contrast to stress hormones, the level of D-dimer was found to be comparable in both groups. D-dimer is the degradation product of fibrinogen and cross-linked fibrin and has increasingly been used in the screening of deep vein thrombosis. Published research studies have documented an elevation in D-dimer levels following fractures of the lower limb [30]. However, the same study fails to demonstrate any impact of pain control measures on the D-dimer level [30].

## Conclusions

The ELETRA approach, offering epidural analgesia for lower limb trauma patients in emergency settings, has proven to be a safe and effective pain management technique. Its implementation is advised wherever practical. Additionally, ELETRA is notable for its capacity to diminish stress responses in patients, highlighting its significant clinical and therapeutic advantages.
